# P366: Alternatives to the biomedical waste incineration in the treatment and waste sorting system in the chain

**DOI:** 10.1186/2047-2994-2-S1-P366

**Published:** 2013-06-20

**Authors:** F Abdoulaye, E Elisante, T Mwinuka, J Katima, J Emmanuel

**Affiliations:** 1 PNUD/FEM, Dakar (Senegal), Tanzania, United Republic of

## Introduction

Within the framework of the International Project UNDP / GEF HCW, which was launched in 2008, the College of Engineering (COET) at the University of Dar es Salaam has developed an affordable technology that is respectful of environment and is based on the concepts of state-of the art autoclave waste treatment systems used in industrialized countries. This equipment without incineration technology has been designed, manufactured and tested at COET through the collaboration of students, teachers, support staff and experts from UNDP / GEF. It is ideal for medium-sized county hospitals of about 100 beds.

## Methods

The equipment includes a horizontal autoclave of 280 liters with a boiler powered by electricity or compressed gas, waste containers made of reusable aluminum (autoclavable), designed to maximize the capacity of the autoclave and eliminate the need to use plastic bags, a waste container support with foot pedals for quick opening, hands-free reusable containers for sharp and pointed waste, horizontal tilt-drop openings, cut-needle containers equipped with cylinder containers and autoclavable syringes, and a hydraulic waste compactor provided with a mechanical press.

## Results

Studies conducted at COE, at Muhimbili National Hospital and the Hospital at CCBRT in Tanzania showed a reduction of 5 log10 of heat-resistant. Geobacillus stearothermophilus spores present in the waste. Waste incineration costs the hospitals of Dar es Salaam from 2000 to 5000 Tanzanian shillings per kilogram. Operating costs for this autoclave are estimated between 100 and 200 shillings/kg including costs for delivery at the landfill.

## Conclusion

In addition to cost savings and avoidance of dioxins and other toxic pollutants from incinerators, autoclave technology that enables the recovery and the second fusion of sterilized materials such as plastic, glass and metal, if latter are sorted in advance. These devices are being produced by manufacturers in Tanzania and Senegal and will be available on the market in 2013.

## Disclosure of interest

None declared

